# Long-Term Clinical Outcomes of Small-Incision Femtosecond Laser-Assisted Intracorneal Concave Lenticule Implantation in Patients with Keratoconus

**DOI:** 10.1155/2022/9774448

**Published:** 2022-03-16

**Authors:** Qi Wei, Hui Ding, Ke Nie, He Jin, Tan Zhong, Hanyang Yu, Zhenduo Yang, Shisi Hu, Linyi He, Xingwu Zhong

**Affiliations:** ^1^State Key Laboratory of Ophthalmology, Zhongshan Ophthalmic Center, Sun Yat-Sen University, Guangdong Provincial Key Laboratory of Ophthalmology and Visual Science, Guangdong Provincial Clinical Research Center for Ocular Diseases, Guangzhou 510060, China; ^2^Hainan Eye Hospital and Key Laboratory of Ophthalmology, Zhongshan Ophthalmic Center, Sun Yat-Sen University, Haikou 570300, China; ^3^Affiliated Hospital of Guilin Medical University, Guilin Medical University, Guilin 541000, China

## Abstract

**Purpose:**

The purpose of this study was to evaluate the long-term prognosis of small-incision femtosecond laser-assisted intracorneal concave lenticule implantation (SFII) in correction of human keratoconus.

**Methods:**

This was a prospective study for 11 patients who received SFII after being diagnosed as progressive keratoconus based on the Amsler–Krumeich classification system. Clinical assessment was performed for all the patients prior to and postsurgically at different time points for 5 years. These included uncorrected distance visual acuity (UDVA), corrected distance visual acuity (CDVA), biomechanically corrected intraocular pressure (bIOP), corneal topography, anterior segment optical coherence tomography (AS-OCT), confocal microscopy, and biomechanical assessment with Corvis ST.

**Results:**

Comparison of preoperative and 60-month postoperative UDVA and CDVA (*P*_60months_=0.081 and 0.001, respectively), all eyes showed an improvement in CDVA. Corneal topography showed no significant changes in corneal anterior K1, K2, posterior K1, K2, posterior elevation, or corneal densitometry compared with preoperative levels (*P* > 0.05). Corvis ST showed that central corneal thickness (CCT) and stiffness at applanation 1 (SP-A1) were significantly greater 1 week postsurgically when compared to the baseline (*P* < 0.05) and remained stable thereafter. The lenticule under the AS-OCT remained transparent throughout the entire postsurgical period. Under confocal microscopy, corneal edema and an increase in cell activation and reflectivity were observed at the lenticule-stromal interface within 1 week postoperatively. These reactions gradually subsided with time within 6 months.

**Conclusion:**

SFII is an effective procedure to prevent the progression of keratoconus due to its minimal invasiveness and capability of maintaining a steady biometry of the cornea.

## 1. Introduction

Keratoconus is a progressive corneal ectasia resulting from localized corneal thinning and steepening. Patients with this condition usually demonstrate irregular astigmatism, an increased aberration, and a deteriorated visual acuity at an age ranging from 9 to 28 years [1]. The prevalence of keratoconus is approximately 0.14%; however, its prognosis is poor if an effective treatment is not available before its deterioration [2]. At present, there are two main treatment methods for keratoconus. One is to slow the progression of the disease, such as corneal collagen cross-linking (CXL), and the other one is to improve vision, such as rigid gas-permeable (RGP) contact lens and intracorneal ring segment implantation (ICRS). Corneal graft is the traditional treatment for advanced keratoconus, which carries a risk of postsurgical infection, stromal opacification, and host-corneal rejection [3]. Additionally, approximately 53% of patients worldwide are unable to receive a corneal transplantation due to lack of corneal grafts [4].

Femtosecond laser has been developed for the correction of refractive errors with minimal invasiveness. It can create both a donor lenticule with expected specifications and a stromal pocket at an expected depth within the host cornea [5]. It has been suggested in some clinical studies that the lenticule from small incision lenticule extraction (SMILE) has a great potential for application in corneal surgery [6, 7]. Femtosecond laser-assisted lenticule implantation has already been used to correct hyperopia in humans [8, 9] and also provides a new surgical approach for the treatment of corneal ulcer [10, 11].

In a previous preliminary study, we have demonstrated that SFII has a faster postoperative recovery than penetrating keratoplasty (PKP) in patients with progressive keratoconus [12, 13]. This study aimed to assess the morphological changes and biomechanical stability of the host cornea in patients with progressive keratoconus over a period of 5 years after SFII.

## 2. Materials and Methods

### 2.1. Study Design

This prospective study was approved by the Ethics Committee of Hainan Eye Hospital at Zhongshan Ophthalmic Center (Sun Yat-sen University, China) and followed the ethical guidelines of the Declaration of Helsinki. A written informed consent was obtained from all patients after the nature of the study, and potential risks were explained (ethics acceptance number: 2016-007).

This prospective study included patients who were diagnosed as stage II to III progressive keratoconus based on the Amsler–Krumeich classification system in one of the eyes [14]. All of the eyes were free of acute inflammation and previous surgery in the anterior segment of the eyes. Inclusion criteria were those who had met the following conditions in the past year: (1) the maximum curvature value of corneal topographic map increased by 0.75 diopters (D) or more; (2) the astigmatism of optometry increased by 0.75D or more, or the spherical equivalent increased by 0.5D or more; (3) the corneal thickness was less than 480 *μ*m; (4) keratoconus apex within the central 2 mm (central keratoconus); (5) physical intolerance to any type of contact lenses [15]. A total 11 eyes of 11 patients (male: 7, female: 4) with the age ranging from 18 to 28 years were recruited to the Hainan Eye Hospital at the ZhongShan Ophthalmic Center between March 2016 and December 2017.

### 2.2. Clinical Examinations

Ophthalmic examinations were performed preoperatively and at different time points postsurgically (1 week and 1, 3, 6, 12, 24, 36, and 60 months). The clinical measurements taken included uncorrected distance visual acuity (UDVA), corrected distance visual acuity (CDVA) and manifest refraction with a synthetical optometer (Nikon, Tokyo, Japan), assessment of ocular anterior segment by a slit-lamp microscope (Topcon, Tokyo, Japan), and corneal photography with a slit-lamp digital image processing system (Topcon, Tokyo, Japan). Corneal topography and anterior chamber evaluation were recorded using the Pentacam HR Scheimpflug camera (OCULUS Optikgeräte GmbH, Wetzler, Germany). Anterior segment optical coherence tomography (Topcon, Tokyo, Japan) and confocal microscopy (HRT III; Heidelberg Engineering GmbH, Heidelberg, Germany) were also performed. The biomechanically corrected intraocular pressure and biomechanical properties of the patients were measured using a Corvis ST system (OCULUS, Wetzlar, Germany).

### 2.3. Surgical Procedures

All donor tissues were collected from the eye bank of the Hainan Eye Hospital in accordance with local guidelines (Hainan Entry-Exit Inspection and Quarantine Bureau of the People's Republic of China). Topical anaesthesia consisted of proparacaine hydrochloride eye drops (Alcaine; Alcon Laboratories, Fort Worth, TX). A myopic correction of −0.75 diopters (28 *μ*m thickness) using a femtosecond laser system under SMILE treatment was performed on the recipient cornea, and the surgical incision was 2.5 mm long. The surgeon unfolded the lenticule with microforceps and created a “stromal pocket” at a depth of 160 *μ*m from the cornea epithelium, followed by implantation of the lenticule into the “stromal pocket.” The surgical procedures were detailed in our previous studies [13, 16]. Following the surgery, all patients received tobramycin and dexamethasone (Alcon Laboratories, US) eye drops 4 times per day for 2 weeks and recombinant bovine basic fibroblast growth factor (Fusion Protein, Bausch and Lomb, Rochester, NY) eye gel 3 times per day for 6 months.

### 2.4. Statistical Analysis

For statistical purposes, UDVA and CDVA data were converted by minimum resolution (logMAR) according to the standard logarithmic visual acuity chart. Statistical analysis was conducted using SPSS 23.0 statistical software (Stanford University, USA). All data that met normal distribution by the Shapiro–Wilk test were expressed as mean ± SD. ANOVA (analysis of variance) with multiple comparisons between groups was compared using LSD pairwise comparison with *P* < 0.05 as statistically significant.

## 3. Results

### 3.1. Visual Acuity and Intraocular Pressure

All 11 patients returned on schedule for their postoperative examinations. The mean values of the manifest sphere went from −9.25 ± 0.47 D at the preoperative level to −8.17 ± 0.52 D at 60 months postsurgically (*F* = 2.744, *P*_60month_=0.159). The mean values of the manifest cylinder went from −6.56 ± 0.76 D at the preoperative level to −6.72 ± 0.61 D at 60 months postsurgically (*F* = 0.091, *P*_60month_=0.771). The preoperative spherical equivalent (SE) was −12.28 ± 1.29 D in the SFII-treated eyes and −11.23 ± 1.26 D at 60 months postsurgically (*F* = 4.718, *P*_60month_=0.066).

**Figure 1:**
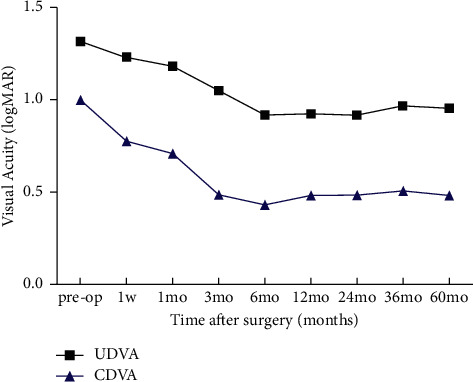
There was no significant change in preoperative UDVA compared with UDVA at 60 months postsurgically (*F* = 3.215, *P*_60months_=0.081). The CDVA showed significant improvement at 3 months postsurgically (*P*_3months_=0.026), and this result remained constant thereafter. (CDVA = corrected distance visual acuity; logMAR = logarithm of the minimum angle of resolution; UDVA = uncorrected distance visual acuity).

The UDVA was found not to have improved (Figure 1) at different time points postsurgically (1 week and 1, 3, 6, 12, 24, 36, 60 months). The CDVA showed improved significantly from 1.00 ± 0.19 to 0.48 ± 0.13 logMAR at 3 months postsurgically (*P*_3month_=0.026), and this result remained constant thereafter. The bIOP elevated rapidly from 11.37 ± 2.32 mmHg to 16.85 ± 3.14 mmHg within 1 week postsurgically (*P*_1week_=0.042) and declined to 12.75 ± 2.18 mmHg at 1 month postsurgically (*P*_1month_=1.000).

### 3.2. Slit-Lamp Microscopy

**Figure 2:**
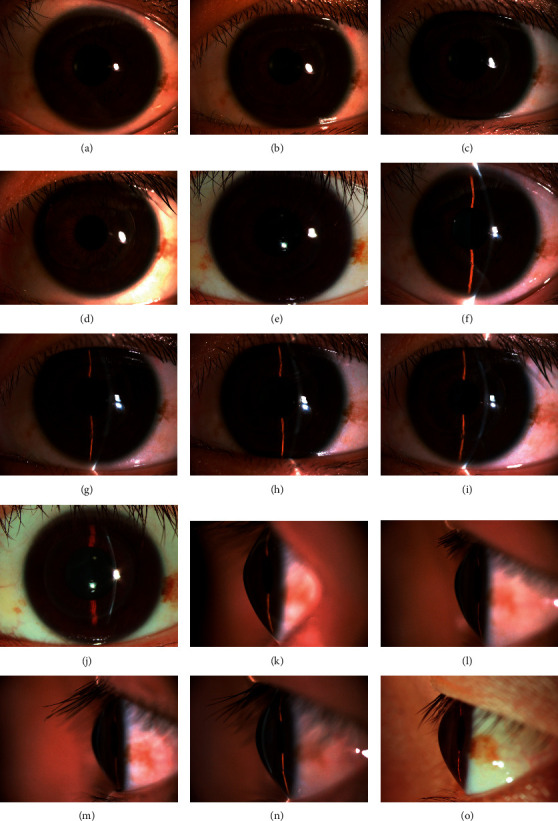
(a, f, k) The slit-lamp microscope images of preoperative patients with cone shaped. (b, g, l) All operated eyes did not show corneal ulceration, corneal neovascularisation, and exudate in the anterior chamber at 1 week postsurgically. (c, h, n) At 1 month postsurgically, slit lamp microscope showed that corneal stromal edema was considerably reduced compared to 1 week postsurgically. (d, i, m) Corneal stromal edema greatly subsided after 3 month postsurgically. (e, j, o) At 60 months postsurgically, a scar line was observed at the periphery of the lenticule, while the central region remained transparent.

All operated eyes did not show corneal ulceration and corneal neovascularisation and exudates in the anterior chamber (Figure 2) throughout the entire follow-up period. At 60 months postsurgically, a scar line was observed at the periphery of the lenticule, while the central region remained transparent.

### 3.3. Pentacam HR Scheimpflug Camera Scanning

**Figure 3:**
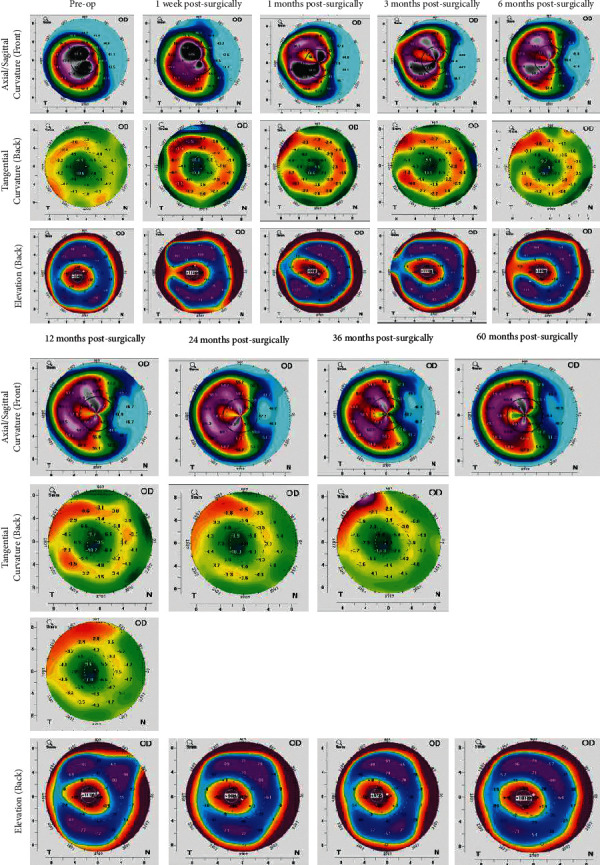
Means of posterior surface curvatures K1 and K2 before the operation were −7.74 ± 0.43 and −8.96 ± 0.48, respectively, and remained stable after surgery. At 60 months postsurgically, the posterior surface curvature K1 and K2 were −7.98 ±k 0.50 and −9.11 ± 0.55, respectively, with no significant change as compared to the preoperative values. The mean of corneal posterior surface height, which is an indicator of surgical stability, was 94.14 ± 13.67 *μ*m preoperatively and 96.01 ± 12.96 *μ*m at 60 months postsurgically, without significant change relative to the preoperative levels.

There were no significant changes in anterior surface curvature K1, K2, posterior surface curvature K1, K2, anterior chamber (AC) depth, AC volume, and posterior surface elevation at different time points postsurgically (1 week and 1, 3, 6, 12, 24, 36, 60 months), as compared to the preoperative results (Table 1). The corneal densitometry (CD) increased within 1 week postsurgically, recovered to the preoperative baseline after 6 months, and remained constant over the following 5 years (*F* = 0.009, *P*_60months_=0.777). Corneal curvature maps demonstrated the stabilization of the corneal surface and posterior elevation after surgery (Figure 3).

### 3.4. Corneal Biomechanical Observation

**Figure 4:**
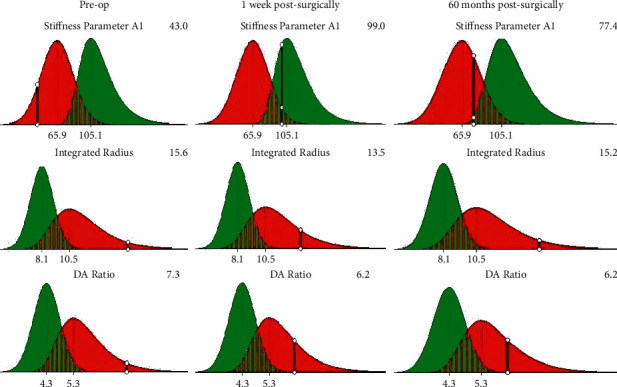
Stiffness parameter at applanation 1 (SP-A1) of the cornea significantly increased from 42.73 ± 8.93 mmHg/mm to 80.08 ± 8.94 mmHg/mm within 1 week postsurgically and remained stable over the following 5 years. Deformation amplitude ratio (DA ratio) did not change significantly from those of the preoperative period (*P*_DAratio_=0.347).

The SP-A1 of the cornea and CCT increased within 1 week postsurgically (F_SPA1_ = 16.666, *P*_SPA1_=0.000, *F*_CCT_ = 24.311, *P*_CCT_=0.000) and remained stable over the following 5 years. The values of the DA ratio and stress-strain index (SSI) did not change significantly at different time points postsurgically (1 week and 1, 3, 6, 12, 24, 36, 60 months). These results were detailed in Table 2 and Figure 4.

### 3.5. AS-OCT Observation

**Figure 5:**
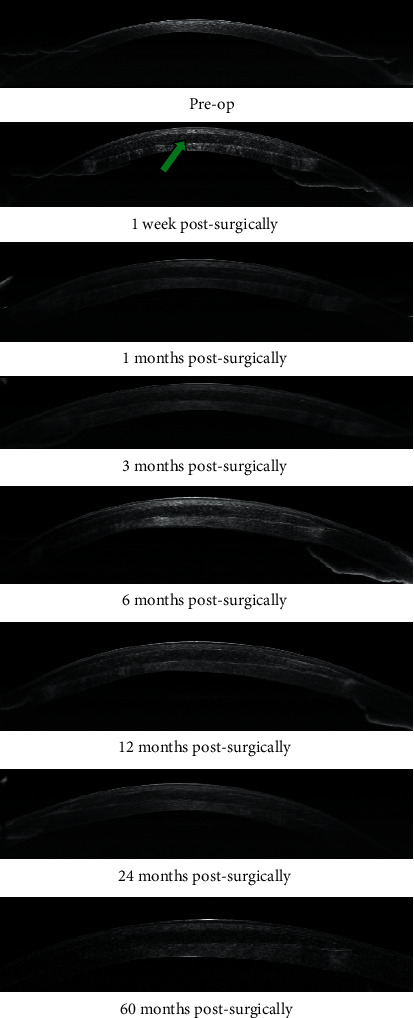
The preoperative images showed that corneal thickness was thin. The corneal thickness of the host increased compared with the preoperative level, and no obvious tissue reaction such as infiltrates, ulcer, and neovascularisation from the host cornea was observed (arrows indicate the lenticule and its location in the “stromal pocket”). Throughout the 60-month observation period, the corneal thickness and transparency of the recipient remained stable.

The lenticule was transparent and well biocompatible within the host cornea at a depth of 160 *μ*m throughout the follow-up in all 11 cases. Keratitis was not observed in any of the host cornea after surgery. The corneal thickness of the host increased compared with the preoperative level, and no obvious tissue reaction, such as infiltrates, ulcer, and neovascularisation, from the host cornea was observed (Figure 5).

### 3.6. Confocal Microscopy

**Figure 6:**
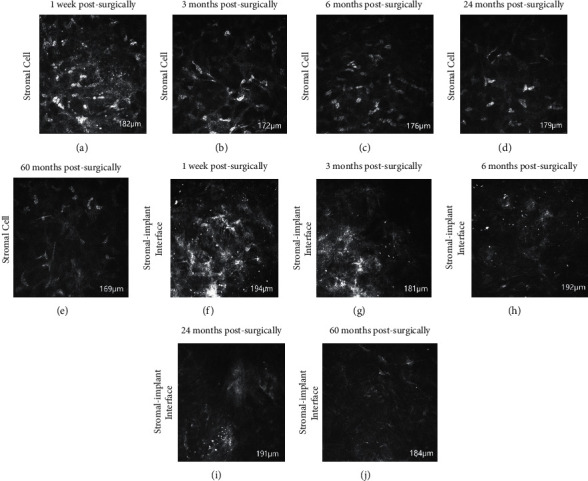
At 1 week postsurgically, there was edema, stromal cell activation at the corneal stroma (a). Lenticule-stroma interfaces are characterized by high reflectivity with bright dots (f). Edema, stromal cell activation and reflectivity gradually decreased over time (3 months postsurgically (b, g); 6 months postsurgically (c, h); 24 months postsurgically (d, i). At 60 months postsurgically, edema subsided and stromal cells remained stable (e), but high reflectivity still appeared in stromal-implant interfaces (j).

At 1 week postsurgically, corneal edema and stromal cell activation were observed in the host cornea (Figure 6(a)) with bright reflected spots and spiky keratocytes along the lenticule-stromal interface (Figure 6(f)). These reactions gradually subsided over time (3 months postsurgically: Figures 6(b) and 6(g); 6 months postsurgically: figures 6(c) and 6(h); 24 months postsurgically: Figures 6(d) and 6(i)). At 60 months postsurgically, edema subsided and stromal cell remained stable (Figure 6(e)), but high reflectivity still appeared in stromal-implant interfaces (Figure 6(j)). Lenticule vascularization, stromal infiltration, and endothelial cell damage were not observed in the cornea throughout the postoperative period.

## 4. Discussion

A successful treatment for keratoconus should be able to stabilize the cornea biologically and mechanically in order to inhibit the progression of corneal ectasias. The human corneal stroma is mainly composed of collagen lamellae, which accounts for approximately 90% of the overall thickness of the cornea [17]. The corneal strength derives from the meshwork of the collagen lamellae, which play a crucial role in the maintenance of normal corneal functions [18, 19]. Our previous animal study has confirmed that the surgically designed “stromal pocket” at a depth of 160 *μ*m from the cornea epithelium facilitates the recovery of the surgical incision of 2.5 mm without damaging the biomechanical stability of the cornea [20, 21].

In this study, all operated eyes achieved a stable condition during the 5-year follow-up period and showed an improvement in CDVA (11 eyes) without progression of ectasia or host corneal rejection. The UDVA at 60 months postsurgically did not deteriorate as compared with the preoperative level, while SE and manifest refraction remained stable from the preoperative level (*P* > 0.05). We observed that biological parameters of the anterior segment were measured by a Pentacam HR, such as anterior and posterior surface K1/K2, AC depth, and posterior surface elevation remain stable after SFII. These results are consistent with a stable UDVA from 1 week to 60 months postsurgically in the present study and from similar patients in previous studies [22]. Vega-Estrada and Alio [23] reported that further deterioration of vision could be prevented if the normal corneal morphology of keratoconus patients was maintained. Greenstein et al. [24] reported that when the biomechanical structure of cornea was enhanced, the higher-order aberrations, corneal topographic index, and subjective visual function of patients with keratoconus could be improved by modest, yet significant.

In this study, bIOP increased rapidly within 1 week postsurgically and returned to preoperative levels at 1 month postsurgically. This short-term bIOP elevation could be related to topical use of corticosteroids, damage to the angle of the anterior chamber, suturing method, and peripheral anterior synechiae in the presence of inflammation [25]. The surgical incision in the present study was 2.5 mm long with no sutures applied. After ruling out surgical factors such as leakage from the surgical incision, angular injury, and suturing method as causes of ocular hypertension, the most likely cause for the elevated bIOP in this study was most likely due to the use of corticosteroids. This hypothesis was further supported by a recovery of the bIOP after the corticosteroid was ceased. In addition, we suspect that another reason may be the reduction of corneal edema and the decline of intraocular pressure caused by the decrease of corneal thickness. Smedowsk et al. [26] reported that there is a medium-strength correlation between bIOP and CCT.

Progression of keratoconus is usually accompanied with an increase in corneal densitometry [27, 28]. Jiménez-García et al. [29] reported that changes in corneal densitometry were consistent with the progression of keratoconus and are one of the indicators for monitoring the deterioration of keratoconus disease. In the present study, we found that the CD value of total corneal thickness increased within 1 week postsurgically, recovered to the preoperative level after 6 months, and remained constant thereafter. This short-term change was likely due to corneal edema after surgery. As the edema subsided, the CD value of total corneal thickness recovered to the preoperative level and remained constant from 6 months to 60 months postsurgically. Although the scarring was observed at the edge of the lenticule through the slit-lamp microscope at 60 months postsurgically, the stable CD value of the total corneal thickness indicates that the transparency of the central area of the lenticules was not significantly affected. During the follow-up period, corneal densitometry remained constant, which indicate the transparency of the cornea remains stable after SFII.

The monitoring of corneal biomechanics is one of the most important indicators in the analysis of changes in the course of keratoconus [30]. It has been shown that corneal thinning in patients with keratoconus reduces corneal stiffness, and this leads to a reduction in the structural integrity of the cornea, which in turn promotes further corneal thinning [31]. Linear regression analysis indicates that SP-A1 is highly accurate in the diagnosis of keratoconus and monitoring of the disease progress [32, 33]. SP-A1 can be used to quantify the resistance of the cornea to deformation, which is the ratio of the displacement between the pressure load on the cornea to the apex of the undeformed cornea and the deflection of the initial flattening [34]. The value of SP-A1 in normal eyes is between 89.32 and 148.95 mmHg/mm [35, 36], which is higher than in keratoconus eyes, ranging from 46.6 to 77.16 mmHg/mm [37, 38]. In the present study, the SP-A1 value was 42.73 ± 8.93 mmHg/mm before surgery. It reached a maximum value of 89.09 ± 10.51 mmHg/mm at 1 month postsurgically and 79.83 ± 6.55 mmHg/mm at 60 months postsurgically. These results indicate that the lenticule implanted increased the corneal strength.

Apart from SP-A1, the DA ratio is highly sensitive and specific in the diagnosis of keratoconus [39]. As corneal stiffness decreases, the less the resistance to deformation, the greater the DA ratio value [40]. We observed a slight decrease in DA ratio from 1 week to 60 months postsurgically, but there was no significant difference compared to the preoperative levels. Hassan et al. [41] found an increase in SP-A1 when looking at corneal biomechanical changes 2 years after corneal collagen cross-linking, but the DA ratio did not change significantly from the preoperative period.

In addition, to eliminate the influence of other factors on corneal biomechanical examination, we observed a new detection index: the SSI. Zhang et al. [42] reported that changes in SSI can be used as an assessment of the effectiveness of treatment in keratoconus. Unlike other stiffness parameters, the change of SSI is not affected by bIOP or CCT and can be used to evaluate the mechanical properties of corneal tissue accurately [43]. Zhao et al. [44] found that the SSI of patients with keratoconus was 0.64 ± 0.12, which was comparable to our preoperative SSI (0.63 ± 0.03). With the implantation of the lenticule, we also found a stable trend of SSI before and at 60 months postsurgically. This suggested that the corneal resistance to deformation had been improved.

To further understand the real-time changes in the recipient cornea, postoperative patients were observed regularly by AS-OCT and confocal microscopy. AS-OCT showed that the corneal thickness of the recipient increased 1 week postsurgically (Figure 5). Confocal microscopy documented that lenticule-stromal interfaces were characterized by high reflectivity with bright dots (Figure 6(f)), and edema, stromal cell activation was observed at the corneal stroma in the 1 week postsurgically (Figure 6(a)). Edema, stromal cell activation and reflectivity gradually decreased over time (3 months postsurgically: Figures 6(b) and 6(g); 6 months postsurgically: Figures 6(c) and 6(h); 24 months postsurgically: Figures 6(d) and 6(i)). At 60 months postsurgically, edema subsided and stromal cell remained stable (Figure 6(e)), but high reflectivity still appeared in stromal-implant interfaces (Figure 6(j)). No evidence of leukocyte infiltration at lenticule-stromal interface was observed during the follow-up. This observation is similar to those of studies by other scholars [45].

We evaluated the morphological changes and biomechanical stability of the host cornea after SFII and demonstrated the potential of this surgical technique for anterior corneal profile restoration. The major limitations of our study include the small sample size, and no effective grouping or comparison of patients with corneal thicknesses >400 *μ*m and ≤400 *μ*m was conducted to observe the different effects of lenticule implantation on patients with different thicknesses.

Our findings suggest that SFII is a safe surgical approach, and stromal lenticule can be well tolerated by the recipient cornea, with a steady state of corneal morphology and biomechanics. It could provide an effective therapeutic approach for patients with progressive keratoconus.

## Figures and Tables

**Table 1:** Postoperative changes in corneal morphology.

Parameters	Time										
	Preop	1wk Postoperatively	1 mo Postoperatively	3 mo Postoperatively	6 mo Postoperatively	12 mo Postoperatively	24 mo Postoperatively	36 mo Postoperatively	60 mo Postoperatively	*F*	*P*_*60mo*_
Anterior, K1, D	51.60 ± 1.79	52.37 ± 2.86	52.27 ± 2.63	51.16 ± 2.31	51.19 ± 2.33	51.14 ± 2.25	50.73 ± 2.13	50.37 ± 2.12	50.11 ± 2.06	0.715	0.677
Anterior, K2, D	58.54 ± 2.47	59.56 ± 2.76	59.76 ± 2.78	59.04 ± 2.81	58.20 ± 2.62	58.40 ± 2.71	57.66 ± 2.65	58.01 ± 2.71	57.24 ± 2.71	1.372	0.233
Posterior, K1, D	−7.74 ± 0.43	−8.16 ± 0.66	−8.31 ± 0.61	−7.94 ± 0.39	−8.04 ± 0.53	−8.04 ± 0.55	−7.96 ± 0.52	−8.04 ± 0.49	−7.98 ± 0.50	1.377	0.231
Posterior, K2, D	−8.96 ± 0.48	−9.39 ± 0.61	−9.43 ± 0.55	−9.19 ± 0.48	−9.16 ± 0.52	−9.18 ± 0.60	−9.10 ± 0.58	−9.21 ± 0.52	−9.11 ± 0.55	1.470	0.193
Elevation, (Back), *μ*m	94.14 ± 13.67	117.00 ± 19.98	100.57 ± 14.29	106.28 ± 14.89	101.57 ± 15.28	99.29 ± 14.95	101.29 ± 13.09	99.28 ± 12.60	96.01 ± 12.96	1.776	0.105
AC volume, mm^3^	190.75 ± 11.03	184.50 ± 12.19	183.25 ± 12.24	185.00 ± 10.43	182.63 ± 11.47	182.88 ± 10.66	184.63 ± 12.45	186.63 ± 13.52	185.38 ± 13.13	0.752	0.646
AC depth, mm	3.58 ± 0.07	3.33 ± 0.09	3.45 ± 0.10	3.50 ± 0.10	3.53 ± 0.10	3.51 ± 0.10	3.54 ± 0.13	3.59 ± 0.14	3.57 ± 0.13	2.79	0.10
Total CD, GSU	15.98 ± 0.57	19.58 ± 0.89	18.98 ± 0.73	17.68 ± 0.68	17.10 ± 0.67	16.21 ± 0.45	15.40 ± 0.22	15.81 ± 0.39	15.87 ± 0.53	0.09	0.777

The anterior K1, DK2; posterior K1, DK2; and elevation back remained stable postoperatively. The *P* value was no statistically significant compared with preoperative levels. D, diopters; K1, minimum central keratometry; K2, maximum central keratometry; AC, anterior chamber; CD, corneal densitometry; GSU, grayscale unit.

**Table 2:** Postoperative changes in corneal biomechanics.

Parameters	Time										
	Preop	1 wk Postoperatively	1 mo Postoperatively	3 mo Postoperatively	6 mo Postoperatively	12 mo Postoperatively	24 mo Postoperatively	36 mo Postoperatively	60 mo Postoperatively	*F*	*P*_*60mo*_
CCT, *μ*m	434.75 ± 14.18	616.75 ± 34.26	622.88 ± 35.84	613.25 ± 34.83	607.50 ± 29.07	617.12 ± 28.03	620.13 ± 31.84	601.25 ± 28.56	610.88 ± 27.40	24.311	0.000
SP-A1, mmHg/mm	42.73 ± 8.93	80.08 ± 8.94	89.09 ± 10.51	84.26 ± 9.28	83.95 ± 9.53	81.56 ± 7.65	76.75 ± 7.01	79.38 ± 7.26	79.83 ± 6.55	16.666	0.000
DA Ratio, mm	6.13 ± 0.39	5.15 ± 0.24	5.40 ± 0.29	5.48 ± 029	5.58 ± 0.32	7.30 ± 2.01	6.77 ± 1.14	6.65 ± 0.72	6.35 ± 0.60	1.159	0.347
SSI	0.63 ± 0.03	0.55 ± 0.03	0.53 ± 0.05	0.47 ± 0.04	0.46 ± 0.05	0.52 ± 0.05	0.53 ± 0.05	0.56 ± 0.06	0.60 ± 0.03	2.580	0.103
bIOP, mmHg	11.37 ± 2.32	16.85 ± 3.14	12.75 ± 2.18	10.87 ± 2.31	11.19 ± 2.11	11.93 ± 1.97	12.03 ± 1.97	12.65 ± 1.89	12.53 ± 1.84	0.480	0.502

CCT, central corneal thickness; SP-A1, stiffness parameter at applanation 1; DA ratio, deformation amplitude ratio; SSI, stress-strain index, bIOP, biomechanically corrected intraocular pressure; bold entries indicate *P* < 0.05.

## Data Availability

The authors confirm that the data supporting the findings of this study are available within the article.
